# Effect of Electroacupuncture on Reuptake of Serotonin via miRNA-16 Expression in a Rat Model of Depression

**DOI:** 10.1155/2019/7124318

**Published:** 2019-12-23

**Authors:** Jun Zhao, Huiling Tian, Hongtao Song, Xu Wang, Tong Luo, Liya Ai, Yumin Fang, Jianghao Zhao, Saiyin Chao-ke-tu, Zhigang Li

**Affiliations:** ^1^School of Acupuncture-Moxibustion and Tuina, Beijing University of Chinese Medicine, Beijing 100029, China; ^2^Inner Mongolia People's Hospital, Hohhot, Inner Mongolia 010010, China; ^3^School of Traditional Chinese Medicine, Beijing University of Chinese Medicine, Beijing 102488, China; ^4^Inner Mongolia International Mongolian Hospital, Hohhot, Inner Mongolia 010065, China; ^5^School of Acupuncture-Moxibustion and Tuina, Beijing University of Chinese Medicine Dongfang College, Langfang 065001, China

## Abstract

The current study aimed to investigate the effects and mechanisms of electroacupuncture (EA) treatment applied to Bai hui (GV20) and Yin tang (GV29) acupoints (1 mA, 2 Hz, continuous wave, 20 minutes) for 28 days in a rat model of chronic unpredictable mild stress (CUMS) on reuptake of serotonin (5-hydroxytryptamine (5-HT)) and miRNA-16 levels in the hippocampus and serum. Rats were housed in individual cages, and CUMS was used to establish a rat model of depression. After EA treatment for 4 weeks, behavioral changes and indices including 5-HT transporter (SERT), 5-HT, and miRNA-16 levels in the hippocampus and serum were examined. The EA treatment significantly improved base levels of sucrose preference and exploratory behavior and significantly decreased SERT protein and mRNA expression in the hippocampus of depressed rats. Significantly increased 5-HT levels were observed, and miRNA-16 levels were significantly decreased in the hippocampus and serum of depressed rats. In conclusion, the antidepressant effects of EA treatment may be affected via inhibition of 5-HT reuptake, upregulation of 5-HT levels, and inhibition of miRNA-16 expression in the hippocampus and serum.

## 1. Introduction

Depression is classified as a mental illness. Clinical symptoms include persistent depression, loneliness, anxiety, cognitive dysfunction, and a high suicide rate, which cause serious economic burden and mental stress to patients and families [1–3]. Meanwhile, data from surveys indicated that 350 million people suffer from depression worldwide, and numbers are increasing [4–6]. The World Health Organization (WHO) predicted that depression will become the primary killer of all global diseases by 2030, threatening human's physical and mental health [7]. Selective serotonin reuptake inhibitors (SSRIs) such as fluoxetine are commonly used to treat patients with depression, but approximately 50% of patients fail to respond to therapy [8]. As such, alternative treatment options may provide novel antidepressant strategies [9].

According to previous studies, Bai hui (GV20) and Yin tang (GV29) are considered primary acupoints for the treatment of depression [10]. The GV20 acupoint has benign regulatory effects on the nervous, endocrine, and immune systems [11, 12]; GV29 also has regulatory effects on the nervous system, causing the release of neurotransmitters such as serotonin (or 5-hydroxytryptamine (5-HT)) [13], which is widely distributed in the central nervous system (CNS) and is known to be associated with depression [14–16]. As a monoamine neurotransmitter and neuromodulator in central information processing, 5-HT has been reported to influence a range of behavioral, physiological, and cognitive functions such as memory, mood, emotions, sleep, and appetite [17]. About 5% of all 5-HT within the body is produced in serotonergic neurons of the CNS and released into synapse [18]. The serotonin transporter (SERT) is located in the membrane of serotonergic axon terminals and is responsible for 5-HT reuptake by serotonergic neurons and its subsequent return to presynaptic terminals, where it is metabolized [19, 20]. The SERT can reuptake and transfer 5-HT into presynaptic neurons from the synaptic cleft of nerve-cell endings, affecting concentration in the synaptic cleft and duration of action of 5-HT, thus effectively terminating signal transmission of 5-HT [21–23]. The main mechanism for clearing extracellular 5-HT after release is SERT-mediated reuptake. In addition, SERT is a target for many widely prescribed antidepressants (e.g., selective serotonin reuptake inhibitors (SSRIs)) and is also a key element in the regulation of central 5-HT function and responsiveness to SSRIs [24]. The SSRI fluoxetine binds SERT with high affinity and acts primarily by blocking SERT at the presynaptic neuron, thus regulating extracellular 5-HT levels and improving emotional status [25–29].

MicroRNAs (miRNAs) are a class of endogenous, noncoding single-stranded microRNAs that are widely found in eukaryotic cells [30, 31]. They can bind to the 3′-untranslated region (UTR) of target genes causing degradation of mRNA and inhibition of protein translation, as well as affecting the expression of target genes [32, 33]. Other studies have reported that some miRNAs are expressed in the brain and play special roles in the proliferation and apoptosis of neurons, which are associated with the development of psychiatric diseases [34–36]. Recent studies have demonstrated that potential miRNA pathway impairments are associated with depression [37]. miRNA-16, particularly, is a posttranscriptional inhibitor of SERT [38]. Studies predicting target genes using in silico computer simulations have revealed that miRNA-16 is complementary to the UTR of SERT mRNA and have validated miRNA-16 as a SERT-targeting miRNA using a luciferase assay [39]. Similar to fluoxetine, miRNA-16 targets SERT and could have antidepressant effects [40]. A previous study on rats reported that chronic treatment with fluoxetine led to increased miRNA-16 levels in serotonergic raphe nuclei and reduced SERT expression, suggesting that miRNA-16 targets SERT [41]. Meanwhile, it has been shown that miRNA-16 expression in the hippocampus is associated with depression-like behavior induced by maternal deprivation during early life [42]. These authors also observed upregulation of miRNA-16 expression in the hippocampus following stressful experiences in early life [42]. Together, these reports suggest miRNA-16 to have a crucial role in SERT function in the hippocampus and stress-related disorders throughout life. However, few studies have focused on serum levels of miRNA-16 in patients with depression. A recent study reported that the level of serum miRNA-16 was significantly decreased in patients with depression, which suggests that miRNA-16 has potential as a high-specificity and high-sensitivity biomarker for depression [43].

The present study aimed to answer the following question: what is the relationship between miRNA-16 and 5-HT in the hippocampus and serum? The mechanism underlying the effect of electroacupuncture (EA) on reuptake of 5-HT and consequent antidepressant effect is thought to be mediated by miRNA-16 but has not been investigated in depth. In the present study, we established a rat model of depression induced by CUMS and assessed and compared the antidepressant effects of fluoxetine and EA. We aimed to elucidate the mechanisms by which EA treatment induced 5-HT reuptake in CUMS model rats and the effects of EA treatment on expression of miRNA-16 in the hippocampus and serum.

## 2. Materials and Methods

### 2.1. Animals

Adult male Sprague-Dawley (SD) rats (8-9 weeks old, body weight of 160–220 g, specific pathogen-free grade) were obtained from Beijing Vital River Laboratories (License number SCXK [Jing] 2016-0006). All rats were housed in a temperature-controlled (22 ± 2°C) and humidity-controlled (35 ± 2%) environment on a 12 h light-dark cycle in a well-ventilated cage with sufficient natural light. All rats were habituated for 7 days. The experimental procedures were approved by the Animal Experimentation Ethics Committee of Beijing University of Chinese Medicine.

### 2.2. Groups

**Figure 1:**
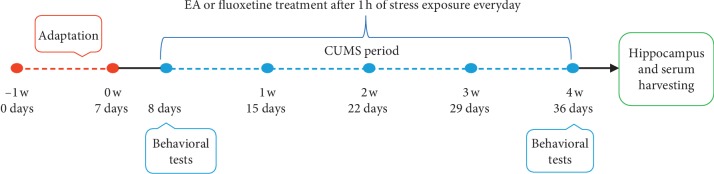
Experimental timeline for chronic unpredictable mild stress.

To ensure consistency of baseline characteristics before experimental procedures were conducted, body weight (BW) was measured, and the sucrose preference test (SPT) and open-field test (OFT) were performed. Seven rats were excluded due to inconsistent baseline characteristics. A total of 48 rats with similar baseline characteristics of BW, SPT, and OFT were randomly divided into four groups (12 rats per group): no-stress stimulation as the control (CON) group, a CUMS group, a fluoxetine (Flu) group, and an EA group. All rats were provided with free access to food and water except during the periods of stress stimuli. Stimulation was performed at 10 a.m. each day. The CUMS, Flu, and EA groups received randomized stress stimulation once a day from day 8 to day 36 for 4 weeks (Figure 1).

### 2.3. Chronic Unpredictable Mild Stress Procedure

After 1 week of acclimatization under animal housing conditions, all groups except the CON group were exposed to CUMS. The CUMS model was established as previously described with minor modifications [44]. In brief, stress stimuli included forced swimming in 4°C water for 5 min, deprivation of either water and/or food for 24 h, inversion for 24 h, tail fastening and clipping for 3 min, cage tilting for 24 h, humid environment for 24 h, and immobilization for 3 h. These stress stimuli were presented for 4 weeks. Each rat in the CUMS model, Flu, and EA groups was exposed to one type of stress stimulus every day followed by housing in individual cages to sustain the depressed state. In contrast, rats in the CON group were housed in two undisturbed cages (six rats per cage). To ensure the unpredictable stress procedure and the same stimulus was not continuously applied, the sequence of stress stimuli was changed randomly every week (Table 1).

### 2.4. Electroacupuncture and Fluoxetine Treatments

**Figure 2:**
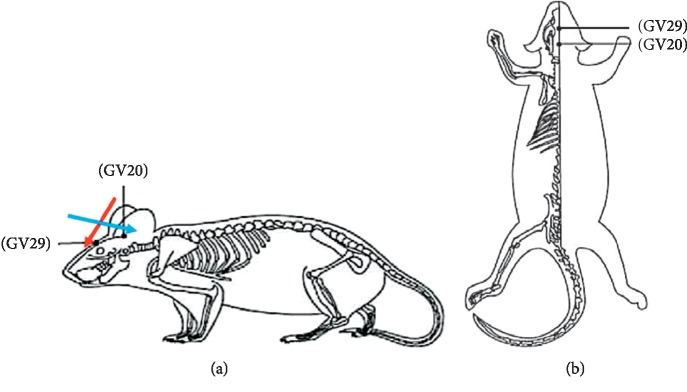
Acupoints diagram of a rat: (a) lateral aspect; (b) superior aspect.

According to the acupoint coordinates, the GV20 (the center of the parietal bone) and GV29 (the middle point of the line between the brow bones) were selected as EA treatment acupoints [45]. The EA method was performed as previously described [10]. The acupuncture needles (one-time use, 0.3 mm in diameter, and 25 mm long; Zhongyan Taihe Medical Instrument Co., Ltd., Beijing, China) were pricked into the skin at GV20 and GV29 to a depth of 5 mm (Figure 2). One needle tip was inserted in an oblique upward direction for GV20 and one in an oblique downward direction for GV29. After twitching slightly, the needle handle was connected to Han's Electroacupuncture Instrument (Beijing Huawei Industry Co., Ltd.). Continuous wave EA was provided at a frequency of 2 Hz and intensity of 1 mA. The stimulation level was maintained at a level where head muscles vibrated slightly and vocalizations were minimized. All rats in the EA group were subjected to EA once a day for 20 minutes during the 4 weeks [46, 47]. Intragastric administration of fluoxetine (2 mg/kg, Lilly Suzhou Pharmaceutical Co., Ltd.) diluted in saline was performed for the Flu group. Treatments were conducted during the same period after 1 h of stress exposure once daily.

### 2.5. Body Weight

The changes in BW compared to normal baseline levels were calculated to evaluate nutritional status and food preference of rats. Body weight was measured on days 8 and 36 for each rat throughout the experimental procedures.

### 2.6. Sucrose Preference Test

Anhedonia was measured based on sucrose preference as previously described, with some modifications [48–50]. Rats were singly housed and trained to drink 1% sucrose solution for 24 h without any water prior to testing. In brief, on the first and last days of stress exposure, rats were subjected to water deprivation for 24 h. Subsequently, rats were given a 24 h window for the sucrose test. One bottle was filled with 1% sucrose solution and the other contained pure water. The position of the two bottles was switched every 12 h. To determine sucrose preference, bottles were preweighed to 250 g. Bottles were placed in individual cages carefully to prevent dripping. All tests were conducted in a quiet room to minimize external disturbances. Sucrose preference was calculated as sucrose intake/(sucrose intake + pure water intake).

### 2.7. Open-Field Test

Locomotor activity was tested in the open field test (OFT), as described previously [51, 52]. Rats were placed in a black, uncovered wooden square area (60 cm × 60 cm × 40 cm). The floor was divided into 25 equal-area squares by legible white lines. The number of times animals crossed the bottom surface sectors (four paws in the same square) was counted as the horizontal index. Exploration with limbs (two front paws raised or climbing the wall) was counted as the vertical index. Rats were placed in the center of the open-field area and allowed 3 minutes of free exploration. After each trial, chambers were cleaned with 75% ethyl alcohol to avoid interference from odors. The OFT was conducted on days 8 and 36.

### 2.8. Immunofluorescence

After anesthesia with 10% chloral hydrate (0.4 mL/100 g), the hippocampus was rapidly removed from the brain. Tissue was placed in 4% paraformaldehyde solution for 12 h and then mixed with 70%, 80%, 95%, and 100% ethanol, xylene, and paraffin at a melting point of 54–56°C for dehydration and embedding. Processed samples were embedded in paraffin and sliced at a thickness of 5-6 *μ*m. Slices were placed in an oven at 40°C for 3 h and washed with phosphate-buffered saline (PBS; 2 min × 3 times). Next, 5% bovine serum albumin (BSA) was added as a block for 20 min. Sequentially, specimens were incubated with diluted primary antibody (anti-SERT, 1 : 100) and washed with PBS (2 min × 3 times) after 1 h in the dark. Specimens were then incubated with fluorescently labeled secondary antibody (CY3, 1 : 200) for 30 min, followed by addition of 4′,6-diamidino-2-phenylindole (DAPI) for 10 min and washing with PBS (2 min × 3 times). Samples were observed under a fluorescence microscope (×200 magnification) after sealing. For each immunofluorescence run, three fields of each slide were randomly selected using ImageJ software. The numbers of positive cells were quantified.

### 2.9. High-Performance Liquid Chromatography

High-performance liquid chromatography (HPLC) equipment included an Agilent 1100 LC High Performance Liquid Chromatograph with a DECADE II SDC electrochemical detector. An Atlantis C18 column (2.1 mm × 100 mm, 3 *μ*m) from Skyam® (Agilent, America). Chromatographic separation was performed using a mobile phase consisting of 0.1 mol/L KH_2_PO_4_ and methanol (10 : 1, v/v; pH = 3) at a flow rate of 0.2 mL/min, column temperature of 30°C, and detection electrode potential of 500 mV.

For hippocampal tissue samples, the first step was to prepare tissue lysis buffer (0.01 g of 0.01% L-cysteine, 0.01 g of 0.5 mmol/L ethylenediaminetetraacetic acid (EDTA)-2Na, and 1.8 mL of 0.2 mol/L HClO_4_) diluted with ultrapure water to 100 mL. The hippocampus of each rat weighed approximately 20–40 mg. Lysis buffer was added to tissue at a ratio of 5 : 1 (v/w); i.e., 50 *μ*L lysate was added to 10 mg tissue. Ultrasonic crushing in an ice bath was performed for 10 seconds for full homogenization, and samples were left for 30 minutes to precipitate. Centrifugation at 15,000 rpm at 4°C for 30 minutes was performed for deproteinization. Finally, 20 *μ*L of hippocampal tissue of the supernatant was injected into the HPLC system for analysis.

For serum samples, serum lysis buffer (0.05 g of 0.01% L-cysteine, 0.1 g of 0.5 mmol/L EDTA-2Na, and 9 mL of 0.2 mol/L HClO_4_) was diluted with ultrapure water to 100 mL. A volume of 250 *μ*L of serum from each rat was collected, and the lysis buffer was added to serum in a ratio of 1 : 5 (v/v); i.e., 10 *μ*L lysis buffer was added to 50 *μ*L serum. Samples were fully homogenized under ice bath conditions and then left for 30 minutes to precipitate. Centrifugation at 15,000 rpm at 4°C for 30 minutes was performed for deproteinization. Finally, 10 *μ*L of clean supernatant from the serum was injected into the HPLC system for analysis.

Quantification of 5-HT was performed using the external standard method. Sample concentrations in the hippocampus or serum were determined from the ratios of their peak areas to corresponding external standards according to the following regression equations: [5-HT] (ng/mg) = (the peak area of 5-HT in hippocampus/690.98), *r* = 0.9999; [5-HT] (ng/*μ*L) = (the peak area of 5-HT in serum/690.98), *r* = 0.9999.

### 2.10. Real-Time Reverse Transcriptase-Polymerase Chain Reaction Analysis of mRNAs

Immediately after anesthesia, 2 mL of whole blood from each rat was collected from the abdominal aorta into a centrifuge tube. Sequentially, blood was settled for 2 h and centrifuged to obtain the upper serum. Hippocampal tissue harvesting was as described previously. All serum and hippocampal tissue samples were stored at −80°C for future analysis. The real-time reverse transcriptase polymerase chain reaction (RT-PCR) technique was used to evaluate the expression of miRNA-16 in the hippocampus and serum, and SERT mRNA in the hippocampus. Total mRNA was isolated from tissue or serum using the QIAzol Lysis Reagent method and quantified by nucleic acid ultraviolet spectrophotometry. First-strand cDNA was synthesized from 2 *μ*L of each mRNA sample with random primers and reverse transcription kits (ABI, America, and Invitrogen, America) for miRNA-16 and SERT, respectively. We conducted PCR conducted using rat-specific forward primer (5′-ATGGTTCGTGCGTAGCAGCACGTAAATATT-3′) and reverse primer (5′-GTCGTATCCAGTGCAGGGTCCGAGGTATTCGCACTGGATACGACCGCCAATA-3′) for miRNA-16 and forward primer (5′-TCGCCTCCTACTACAACACC-3′) and reverse primer (5′- ATGTTGTCCTGGGCGAAGTA-3′) for SERT. To normalize miRNA-16 and SERT data for quantitative analysis, we performed RT-PCR on the same cDNA sample using forward primer (5′-GCTTCGGCAGCACATATACTAAAAT-3′) and reverse primer (5′-CGCTTCACGAATTTGCGTGTCAT-3′) for U6 as the internal reference and forward primer (5′-CAACTCCCTCAAGATTGTCAGCAA-3′) and reverse primer (5′-GGCATGGACTGTGGTCATGA-3′) for glyceraldehyde 3-phosphate dehydrogenase (GAPDH). We used the SYBR Green real-time PCR Master Mix (Roche, Japan) for real-time PCR to evaluate the abundance of PCR products. Relative expression of target mRNA was calculated using the 2^−ΔΔCT^ formula, normalized against internal endogenous GAPDH reference gene for the same sample. The data of other groups are expressed as multiples of the control group.

### 2.11. Statistical Analysis

Data analysis was performed using SPSS (ver 22.0, IBM) statistical software package. All data are expressed as mean ± standard error ($\bar{x}\pm s$). Body weight gain, sucrose consumption changes, vertical or horizontal scores, and aimed indices were analyzed using one-way analysis of variance (ANOVA) followed by the least significant different (LSD) test to evaluate intergroup differences. We considered *P* values less than 0.05 to be statistically significant.

## 3. Results

### 3.1. Electroacupuncture Treatment Improved Behavioral Indices of Depression after Chronic Unpredictable Mild Stress

**Figure 3:**
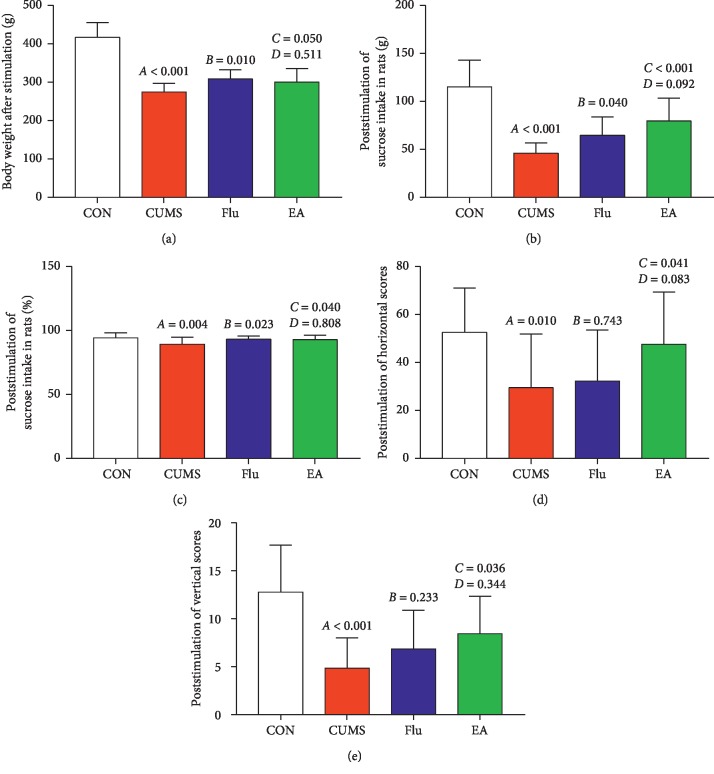
Effects of electroacupuncture treatment on behavioral indices of depression after chronic unpredictable mild stress: (a) differences in body weight following electroacupuncture (EA) treatment (*F* = 49.514; *P* < 0.001); (b) differences in sucrose intake levels following EA treatment poststimulation (*F* = 22.431; *P* < 0.001); (c) differences in sucrose preference index following EA treatment poststimulation (*F* = 3.420; *P*=0.025); (d) differences in horizontal scores following EA treatment poststimulation (*F* = 3.470; *P*=0.024); (e) differences in vertical scores following EA treatment poststimulation (*F* = 8.271; *P* < 0.001). Differences are presented as follows: A, chronic unpredictable mild stress (CUMS) compared with the control (CON); B, fluoxetine treatment (Flu) compared with CUMS; C, EA compared with CUMS; D, EA compared with Flu. Abbreviations: CON, control; CUMS, chronic unpredictable mild stress; EA, electroacupuncture; Flu, fluoxetine.

To investigate the effects of EA on depression-like behaviors in a rat model of depression induced by CUMS, we compared BW, SPT, and OFT pre- and poststimulation. There are no significant differences in BW, water intake, sucrose intake, sucrose preference index, or horizontal and vertical scores between groups prestimulation (*P* > 0.05). However, after stress stimulation for 4 weeks, compared with rats in the CON group, rats in the CUMS group had lower BW, sucrose intake, sucrose preference index, and horizontal and vertical scores (all *P* < 0.01, Figures 3(a)–3(e)). Compared with the CUMS group, the EA treatment group had significantly increased BW, sucrose intake, sucrose preference index, and horizontal and vertical scores (*P* < 0.05, *P* < 0.01, *P* < 0.05, *P* < 0.05, and *P* < 0.05, respectively; Figures 3(a)–3(e)). Similarly, increases in BW, sucrose intake, and sucrose preference index were observed in the Flu treatment group compared with the CUMS group (*P* < 0.01, *P* < 0.05, and *P* < 0.05, respectively; Figures 3(a)–3(c)). No significant differences were observed in any representative numerical index between EA and Flu groups.

### 3.2. Effects of Electroacupuncture Treatment on Serotonin Transporter Expression

**Figure 4:**
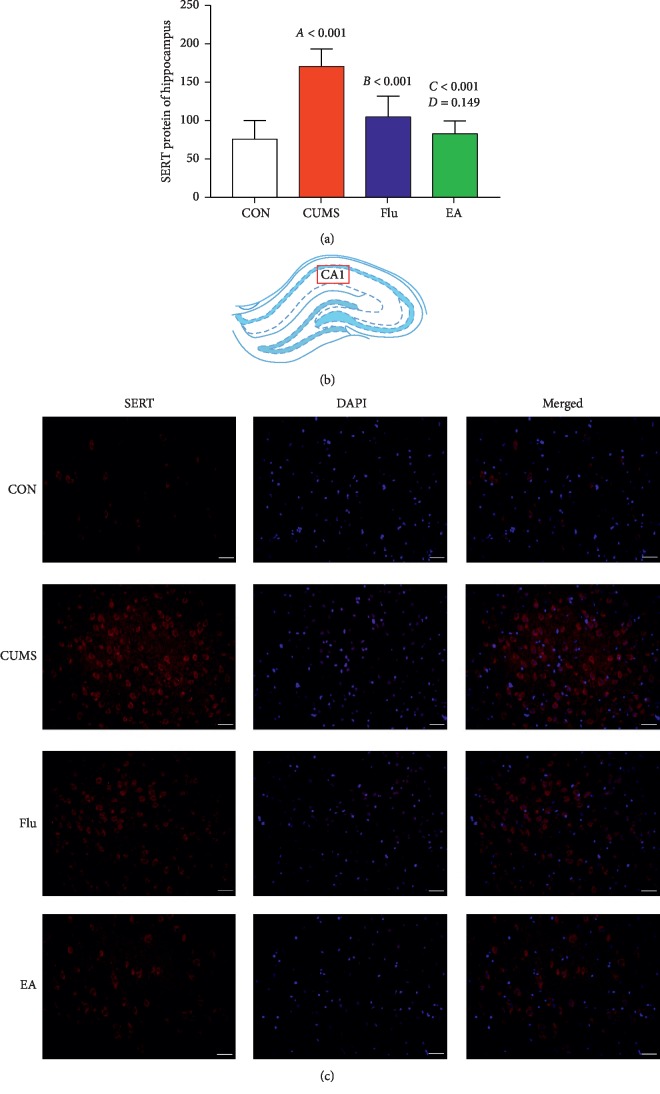
(a) Expression of serotonin transporter (SERT) protein in the hippocampus using a histogram (*F* = 17.449; *P* < 0.01). A, chronic unpredictable mild stress (CUMS) compared with the control (CON); B, fluoxetine treatment (Flu) compared with CUMS; C, electroacupuncture (EA) compared with CUMS; D, EA compared with Flu. (b) Hippocampus CA1 was selected for observation. (c) Expression of SERT protein in the hippocampus CA1 in representative fluorescent images. Red fluorescence indicates SERT-protein-positive cells. Scale bar, 20 *μ*m. Abbreviations: CON, control; CUMS, chronic unpredictable mild stress; DAPI, 4′,6-diamidino-2-phenylindole; EA, electroacupuncture; Flu, fluoxetine; SERT, serotonin transporter.

Immunofluorescence analyses revealed that the level of SERT protein in the hippocampus of each group was significantly altered after CUMS for 4 weeks. Compared with the CON group, the CUMS group has higher SERT expression in the hippocampus (*P* < 0.01). Treatment with EA resulted in an obvious decrease in SERT expression compared with that in the CUMS group (*P* < 0.01). Similarly, the level of SERT in the hippocampus is significantly reduced (Flu versus CUMS: *P* < 0.01) (Figures 4(a)–4(c)).

### 3.3. Effects of Electroacupuncture Treatment on Serotonin Levels

**Figure 5:**
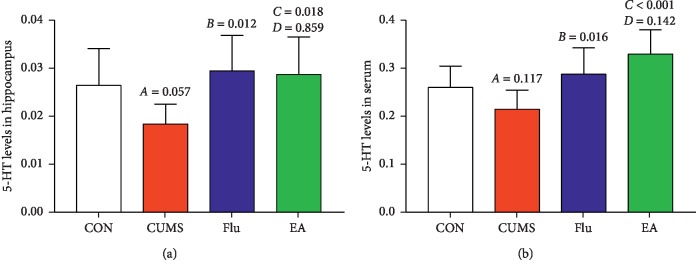
Differences in serotonin (5-HT) levels in the (a) hippocampus (*F* = 3.218; *P*=0.045) and (b) serum (*F* = 6.111; *P*=0.004) following electroacupuncture treatment. A, chronic unpredictable mild stress (CUMS) compared with the control (CON); B, fluoxetine treatment (Flu) compared with CUMS; C, electroacupuncture (EA) compared with CUMS; D, EA compared with Flu. Abbreviations: 5-HT, serotonin; CON, control; CUMS, chronic unpredictable mild stress; EA, electroacupuncture; Flu, fluoxetine.

**Figure 6:**
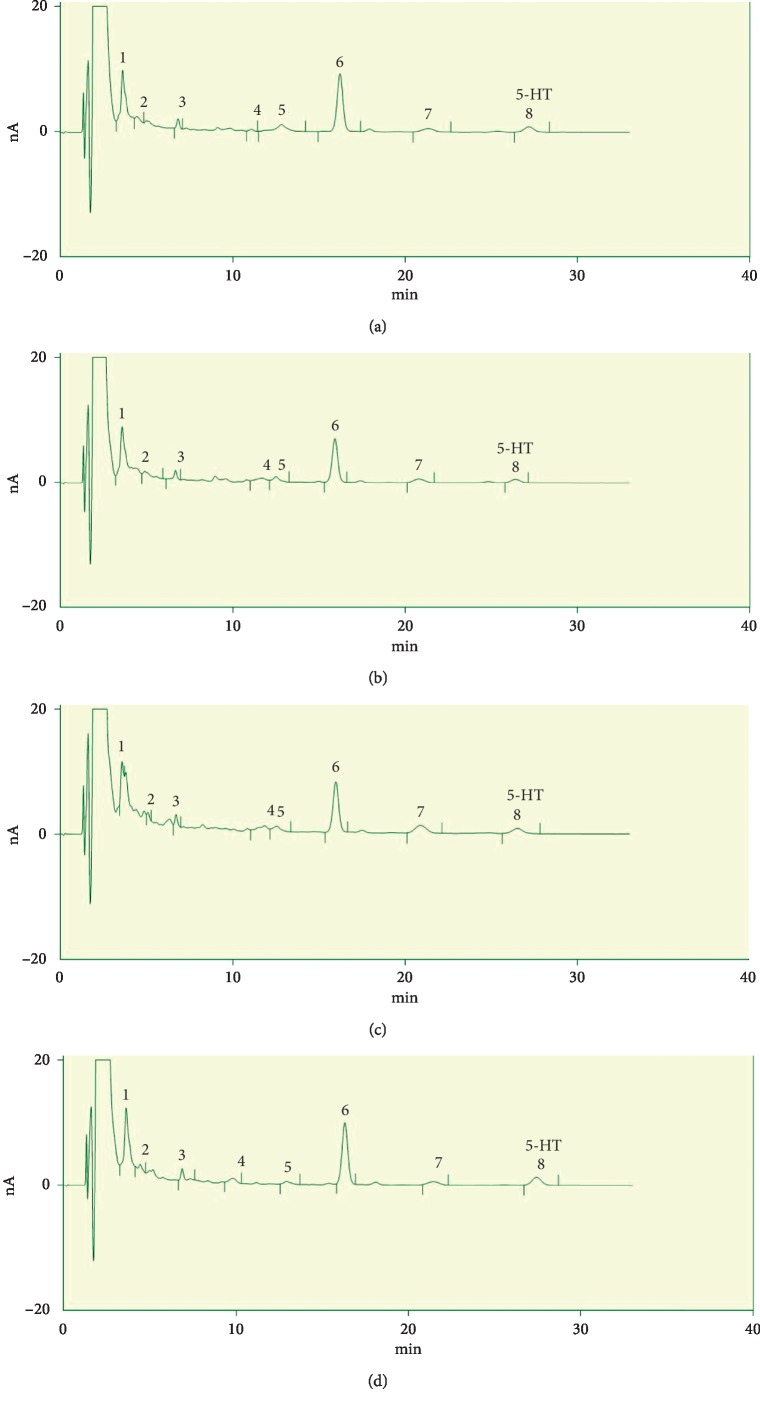
Differences in hippocampal serotonin levels: (a) CON group; (b) CUMS group; (d) EA group; (c) Flu group. Abbreviations: 5-HT, serotonin; CON, control; CUMS, chronic unpredictable mild stress; EA, electroacupuncture; Flu, fluoxetine.

**Figure 7:**
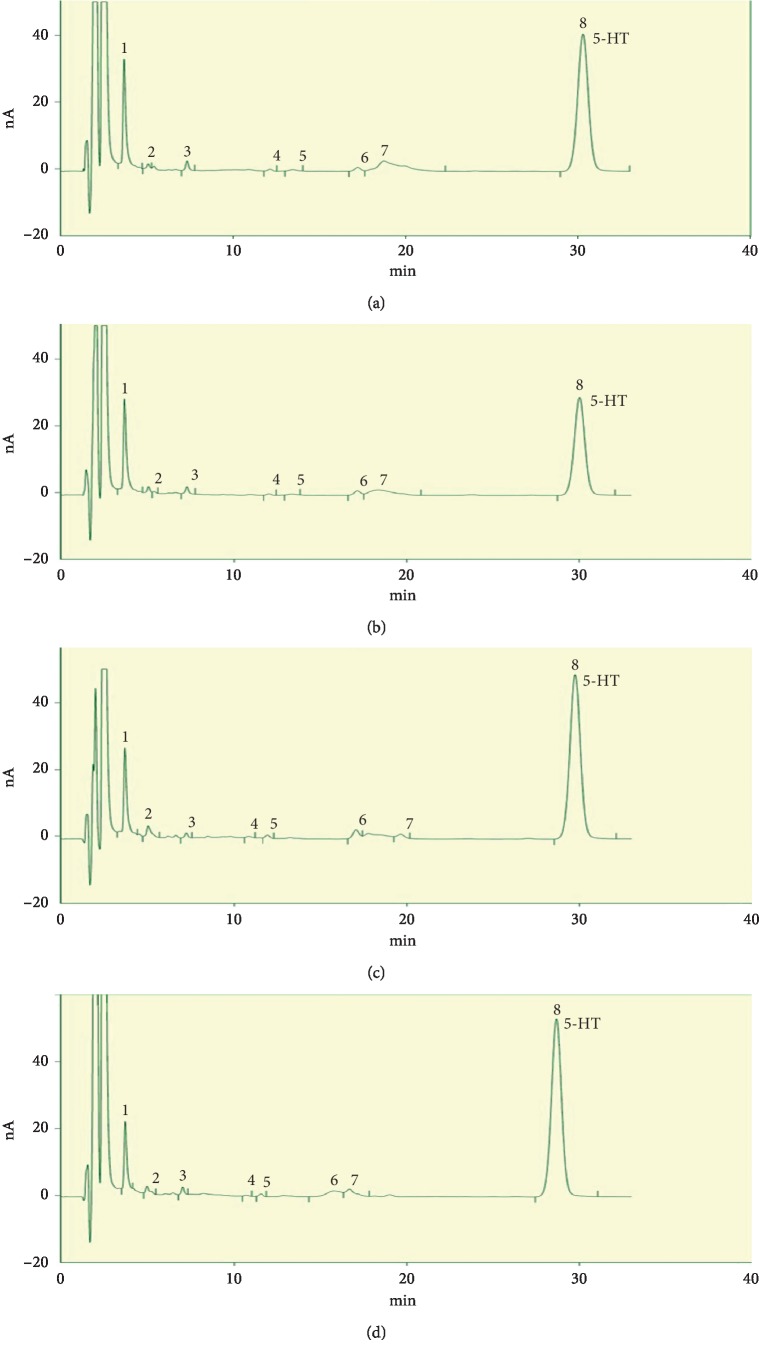
Differences in serum serotonin levels: (a) CON group; (b) CUMS group; (d) EA group; (c) Flu group. Abbreviations: 5-HT, serotonin; CON, control; CUMS, chronic unpredictable mild stress; EA, electroacupuncture; Flu, fluoxetine.

Hippocampal 5-HT levels were significantly increased in Flu and EA versus CUMS (*P* < 0.05 and *P* < 0.05, respectively; Figure 5(a)). Compared with CON rats, those receiving EA had higher 5-HT serum levels (*P* < 0.05; Figure 5(b)). Similarly, 5-HT in serum was significantly increased in the Flu and EA groups versus the CUMS group (*P* < 0.05 and *P* < 0.01, respectively; Figure 5(b)). However, no significant differences in 5-HT were observed in the hippocampus or serum of EA versus Flu groups. The HPLC chromatograms of hippocampal samples and serum samples are depicted in Figures 6(a)–6(d) and 7(a)–7(d), respectively.

### 3.4. Effect of Electroacupuncture Treatment on Expression of Serotonin Transporter mRNA and miRNA-16

**Figure 8:**
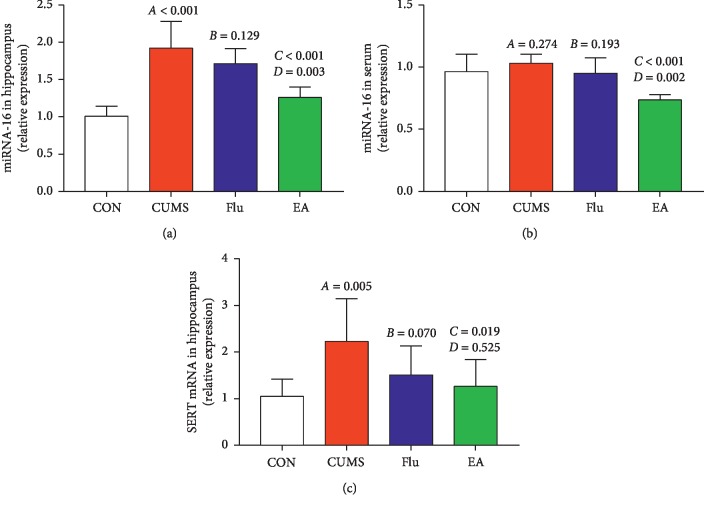
Expression levels of miRNA-16 and serotonin transporter mRNA: (a) relative miRNA-16 expression in the hippocampus (*F* = 19.973; *P* < 0.01); (b) relative miRNA-16 expression in serum (*F* = 9.000; *P*=0.001); (c) relative serotonin transporter mRNA expression in the hippocampus (*F* = 3.713; *P*=0.028). A, chronic unpredictable mild stress (CUMS) compared with the control (CON); B, fluoxetine treatment (Flu) compared with CUMS; C, electroacupuncture (EA) compared with CUMS; D, EA compared with Flu. Abbreviations: CON, control; CUMS, chronic unpredictable mild stress; EA, electroacupuncture; Flu, fluoxetine; SERT, serotonin transporter.

Compared with the CON group, the CUMS group had higher levels of miRNA-16 (*P* < 0.01; Figure 8(a)) and SERT mRNA in the hippocampus (*P* < 0.01; Figure 8(c)). miRNA-16 levels in the hippocampus and serum are significantly reduced in the EA group compared to those in the CUMS group (*P* < 0.01 and *P* < 0.01, respectively; Figures 8(a) and 8(b)). Levels of SERT mRNA in the hippocampus are significantly reduced in the EA group compared with the CUMS group (*P* < 0.05; Figure 8(c)). Levels of miRNA-16 in the hippocampus and serum were significantly reduced in the EA group compared with the Flu group (*P* < 0.01 and *P* < 0.01, respectively; Figures 8(a) and 8(b)). The level of SERT mRNA in the hippocampus was not significantly different (EA versus Flu).

### 3.5. Correlation Analysis

**Figure 9:**
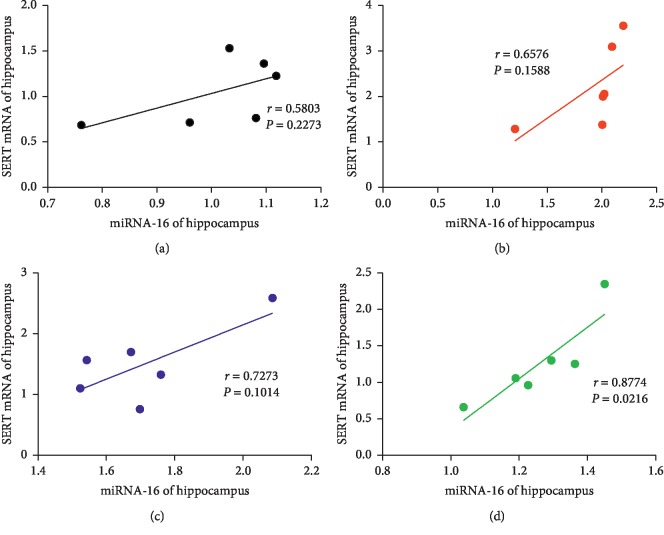
Correlations of miRNA-16 and SERT mRNA in the hippocampus in the (a) CON group, (b) CUMS group, (c) Flu group, and (d) EA group. Abbreviations: CON, control; CUMS, chronic unpredictable mild stress; EA, electroacupuncture; Flu, fluoxetine; SERT, serotonin transporter.

**Figure 10:**
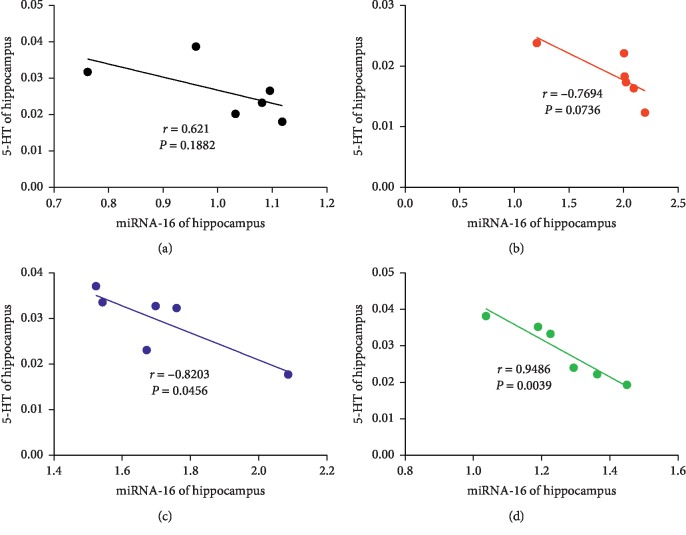
Correlations of miRNA-16 and serotonin in the hippocampus in the (a) CON group, (b) CUMS group, (c) Flu group, and (d) EA group. Abbreviations: CON, control; CUMS, chronic unpredictable mild stress; EA, electroacupuncture; Flu, fluoxetine.

**Figure 11:**
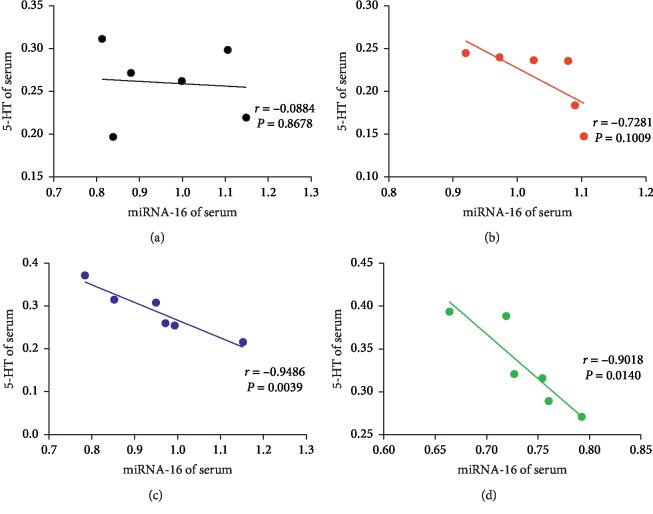
Correlations of miRNA-16 and serotonin (5-HT) in the serum in the (a) CON group, (b) CUMS group, (c) Flu group, and (d) EA group.

Correlation analysis was used to analyze the relationship between miRNA-16 and SERT mRNA in the hippocampus, miRNA-16 and 5-HT in the hippocampus, and miRNA-16 and 5-HT in serum. A positive correlation was observed between miRNA-16 and SERT mRNA levels in the EA group (*r* = 0.8774; *P*=0.0216) (Figure 9(d)). Moreover, a negative correlation was observed between miRNA-16 and 5-HT in the hippocampus in the EA group (*r* = −0.9486; *P*=0.0039) (Figure 10(d)); similarly, a negative correlation was also observed between miRNA-16 and 5-HT in the hippocampus in the Flu group (*r* = −0.8203; *P*=0.0456) (Figure 10(c)). The miRNA-16 and 5-HT levels in serum in the Flu group were negatively correlated (*r* = −0.9486; *P*=0.0039) (Figure 11(c)); similarly, a negative correlation was observed between miRNA-16 and 5-HT serum levels in the EA group (*r* = −0.9018; *P*=0.0140) (Figure 11(d)).

## 4. Discussion

Depression is generally considered to be a multisystem disease with complex pathophysiology involving different signaling pathways. As a neurotransmitter, 5-HT, is known to be associated with depression. The posttranscriptional inhibitor of SERT, miRNA-16, is a key regulator of 5-HT signaling. In the present study, we analyzed the 5-HT and miRNA-16 levels of rats exposed to CUMS after a 4-week EA treatment. Hippocampus and serum were analyzed due to their involvement in the physiological and emotional changes associated with depression.

Presently, there are two common methods to assess the differences in antidepressant effects between acupuncture and fluoxetine when using CUMS to establish a rat model of depression. Method 1: (1) build the depression model by CUMS; (2) 3–6 weeks later, select successful modeling rats and randomly divide them into the CUMS group and medication administration group; and (3) evaluate the antidepressant effects of medicine by comparing the depression-relevant behaviors at pre- and postintervention. Method 2: (1) utilizing fluoxetine and acupuncture during CUMS can prevent depression-like behavioral changes; (2) after the modeling, evaluate the effects of intervention by detecting the depression-like behaviors of animal in each group. The CUMS is employed to mimic negative life events which human beings are facing and to produce depressive-like behaviors in rats, as the key point of CUMS lies in the selection of unpredictable stressors. However, the influence of different time periods, different environments, and other factors on animal behaviors on the first method cannot be avoided. In our research, the rats were randomly exposed to unpredictable stressors which are similar to the experiences of patients with depression. As is known to all, the depressed patients cannot completely get rid of unpredictable stress stimulation when they receive treatment. Hence, the intervention of rats while receiving CUMS stimulation are in line with the practice of clinical research. The present study used measurements of body weight, sucrose preference test (SPT), and open-field test (OFT) to assess the behavior of rats after CUMS-induced depression, including the growth and metabolism of the rats, the sensitivity to reward stimuli, and ability to adapt to a new environment. Compared with the CON group, our findings indicate that CUMS caused poor appetite, slow weight gain, insensitivity to reward stimuli, and reduced exploration in rats. Therefore, we believed that the rats in the CUMS group have showed depression-like behaviors, and CUMS has been successfully set up [53, 54]. Furthermore, we detected the effects of EA or fluoxetine on the rats after the 4-week experimental procedures to predict its antidepressant efficacy by evaluating the behavioral parameters. Compared with the CUMS group, our results showed that fluoxetine or EA administration during the CUMS period could restore depression-like behaviors in rats induced by chronic mild unpredictable mild stress. And it is similar to the previous basic report which suggested that chronic fluoxetine administration during CUMS can prevent depression-like behaviors [55]. Therefore, we can conclude that EA not only has antidepressant effects but could also prevent depression-like behaviors of rats in building modules of depression.

Traditional Chinese Medicine (TCM) believes that Bai hui (GV20) and Yin tang (GV29) are the main acupoints of the Du Meridian. The Du Meridian is closely related to the internal organs, and thus it can reconcile the five internal organs' spirit and emotion to coordinate the effects of yin and yang. The GV20 and GV29 acupoints are considered to be effective acupoint modules for the treatment of depression [52]. Clinical findings have demonstrated the ameliorative effects of EA therapy for depression [10]. Our previous studies have suggested that EA at GV20 and GV29 acupoints increases 5-HT levels in the hippocampus leading to improvements in behavioral parameters of depression in rats [56, 57]. Fluoxetine is an SSRI which acts by blocking the 5-HT transporter (SERT) at the presynaptic neuron to regulate extracellular 5-HT levels and improve emotional status [29]. Fluoxetine has been shown to act on 5-HT neurons in the raphe, in turn leading to the reduced expression of miRNA-16 in the hippocampus [58]. Treatment with fluoxetine has also been found to rescue hippocampal miRNA-16 expression [59]. However, the effect of EA on miRNA-16 expression has not been investigated in depth. Furthermore, whether EA affects the reuptake of 5-HT or expression of its transporter is still unclear.

It has been shown that various miRNAs are expressed highly in the central nervous system, representing a powerful mechanism of regulating the protein content of neuronal cells [16]. Thus, some miRNAs are expressed in the brain and play vital roles in brain functions such as the proliferation and apoptosis of neurons, which are associated with psychiatric diseases [34–36]. In rats subjected to maternal deprivation, higher expression of miRNA-16 was observed in the hippocampus relative to the control during early life [42]. On the contrary, the research also reported that chronic unpredictable stress-induced depressive behaviors exhibited no association with hippocampal miRNA-16 expression [42]. In addition, miRNAs exist in the peripheral blood in a partially stable form. Therefore, these molecules may represent potential blood biomarkers [20]. One study indicated that serum levels of miRNA-16 are increased in rats after CUMS [60]. In contrast, clinical reports revealed that serum levels of miRNA-16 are reduced in patients with depression [43]. In the present study, we found the disease model caused overall upregulated miRNA-16 levels in the hippocampus but did not significantly affect serum miRNA-16 levels. However, it is worth noting that the antidepressive effects of EA on alleviating sucrose intake levels and the horizontal and vertical scores were more compelling than CUMS. We found that EA could reverse the upregulated expression levels of miRNA-16 in the hippocampus induced by CUMS and inhibit the expression of serum miRNA-16. All these results might indicate that the antidepressive response of EA is related to miRNA-16. Based on the data from above studies, we can conclude that there are differences in miRNA-16 expression in different models of depression. To date, there is no universally recognized biomarker for experimental depression, partially due to the difficulties in accessing brain tissue. Serum miRNA-16 might be used as a potential target for the treatment of depression. Furthermore, future studies would be to further explore its diagnostic value.

There have been studies investigating the relationship of 5-HT with mood disorders. In the CNS, 5-HT acts as a neurotransmitter in the dorsal raphe nucleus of the brain, with an important role in the regulation of cognitive, mood, behavioral, physiological, and neuroendocrine functions [17]. Depression is caused by insufficient 5-HT. Clinical research has reported a reduction in 5-HT release in the hippocampus of patients with depression [61, 62]; however, only a few studies have investigated peripheral 5-HT in such patients. Previous studies have pointed out that peripheral 5-HT does not cross the blood-brain barrier (BBB) and the central 5-HT has no effect on the periphery [63]. In contrast, one recent study has suggested that SERT expression on BBB's largest soft membrane vessels is a potential mechanism for peripheral 5-HT access to the CNS [64]. An early study reported that serum 5-HT may be a biomarker for auxiliary diagnosis of depression [65], while another reported decreased serum levels of 5-HT in postpartum depression to be associated with 5-HT metabolism in the postpartum period [66]. Thus, we further studied the effects of EA on 5-HT levels in peripheral serum and central hippocampus of rats and consequent antidepressant effects.

The results of our study show the level of 5-HT to be significantly decreased in the CUMS group, but EA caused upregulation of 5-HT in the hippocampus and serum, in line with the above conclusion that elevation of 5-HT may be associated with antidepressive effects.

Data from laboratory experiments have provided compelling evidence that miRNA-16 is complementary to the 3′-UTR of SERT mRNA and exerts strong regulatory effects on SERT expression, which is associated with depression [39]. Recent studies have shown that SERT-targeting miRNA-16 could be involved in relaying the antidepressant effects of SSRIs [40, 67]. Although we discussed the expression of miRNA-16 in the hippocampus or serum in CUMS models, the relationship between miRNA-16 and SERT mRNA has not been directly investigated.

Moreover, based on the causes of decreased 5-HT levels in the hippocampus and serum of rats in a depression model, the present study explored how the SERT transporter could reuptake 5-HT and potential involvement of miRNA-16 which affects the transmission of 5-HT signaling and may be related to depression. Whether EA achieved these effects by inhibiting 5-HT reuptake remains unclear. During exploring the effect of EA treatment on 5-HT reuptake, we further examined the expression of SERT protein and mRNA, which revealed that CUMS can induce the protein and mRNA expression of SERT in hippocampus. Conversely, EA treatment on rats resulted in downregulation of SERT protein levels and SERT mRNA in the hippocampus, thus decreasing 5-HT reuptake and leading to increased 5-HT levels in CUMS-induced depressed rats. Similarly, the expression of SERT protein and mRNA in the hippocampus was significantly decreased after Flu treatment. Correlation analysis was used to analyze the relationship between miRNA-16, SERT mRNA, and 5-HT. Interestingly, a positive correlation between miRNA-16 and SERT mRNA was observed in the hippocampus after EA treatment. Conversely, negative correlations between miRNA-16 and 5-HT in the hippocampus were observed after Flu and EA treatment. Similarly, after treatment with EA and Flu, miRNA-16 and 5-HT in serum were negatively correlated. The statistically significant data show that miRNA-16 may be directly associated with 5-HT reuptake.

Based on these results, the antidepressant efficacy of EA and Flu treatment may be achieved via inhibition of 5-HT reuptake in the hippocampus and serum following CUMS as well as upregulation of 5-HT levels. Fluoxetine regulates SERT protein expression by miRNA-16, and SERT in turn regulates 5-HT synthesis and transmission. Compared with fluoxetine, EA treatment caused significant inhibition of the expression of SERT mRNA and SERT protein. The EA treatment significantly downregulated miRNA-16 levels in the hippocampus and serum; this inhibition was more obvious than the regulation by fluoxetine. Therefore, we believe that the antidepressant mechanisms of EA may occur via inhibition of target genes by miRNA-16. miRNA-16 and its target SERT mRNA may provide an experimental basis to guide clinical treatment. The limitation of this study is that we did not compare the complementary antidepressant mechanisms of EA and fluoxetine. Our results provide an experimental basis for the combined application of the two treatments.

## 5. Conclusions

Treatment with EA could restore depression-like behaviors in rats induced by chronic mild unpredictable mild stress. This treatment inhibited the expression of SERT protein via miRNA-16, which further inhibited the reuptake of 5-HT resulting in near-normal levels of miRNA-16 and 5-HT in the hippocampus and serum.

## Figures and Tables

**Table 1:** Daily stressful events in the chronic unpredictable mild stress rat model.

Days	Monday	Tuesday	Wednesday	Thursday	Friday	Saturday	Sunday
W1	Food deprivation, 24 h	Swimming in 4°C, 5 min	Fastened and clipped tail, 3 min	Inversion	Cage tilting, 24 h	Humid environment, 24 h	Immobilization, 3 h
W2	Swimming in 4°C, 5 min	Cage tilting, 24 h	Water deprivation, 24 h	Fastened and clipped tail, 3 min	Inversion	Immobilization, 3 h	Humid environment, 24 h
W3	Food deprivation, 24 h	Inversion	Immobilization, 3 h	Humid environment, 24 h	Cage tilting, 24 h	Fastened and clipped tail, 3 min	Swimming in 4°C, 5 min
W4	Water deprivation, 24 h	Cage tilting, 24 h	Swimming in 4°C, 5 min	Humid environment, 24 h	Inversion	Immobilization, 3 h	Fastened and clipped tail, 3 min
